# Interactions of Insula Subdivisions-Based Networks with Default-Mode and Central-Executive Networks in Mild Cognitive Impairment

**DOI:** 10.3389/fnagi.2017.00367

**Published:** 2017-11-09

**Authors:** Ganesh B. Chand, Junjie Wu, Ihab Hajjar, Deqiang Qiu

**Affiliations:** ^1^Department of Medicine, Emory University School of Medicine, Atlanta, GA, United States; ^2^Department of Radiology and Imaging Sciences, Emory University School of Medicine, Atlanta, GA, United States; ^3^Department of Neurology, Emory Alzheimer’s Disease Research Center, Emory University School of Medicine, Atlanta, GA, United States; ^4^Department of Biomedical Engineering, Emory University and Georgia Institute of Technology, Atlanta, GA, United States

**Keywords:** central-executive network, dynamical causal modeling, default mode network, insula subdivisions, insula-based network

## Abstract

Interactions between the brain networks and subnetworks are crucial for active and resting cognitive states. Whether a subnetwork can restore the adequate function of the parent network whenever a disease state affects the parent network is unclear. Investigations suggest that the control of the *anterior insula-based network* (AIN) over the default-mode network (DMN) and central-executive network (CEN) is decreased in individuals with mild cognitive impairment (MCI). Here, we hypothesized that the *posterior insula-based network* (PIN) attempts to compensate for this decrease. To test this, we compared a group of MCI and normal cognitive individuals. A dynamical causal modeling method has been employed to investigate the dynamic network controls/modulations. We used the resting state functional MRI data, and assessed the interactions of the AIN and of the PIN, respectively, over the DMN and CEN. We found that the greater control of AIN than that of DMN (Wilcoxon rank sum: *Z* = 1.987; *p* = 0.047) and CEN (*Z* = 3.076; *p* = 0.002) in normal group and the lower (impaired) control of AIN than that of CEN (*Z* = 8.602; *p* = 7.816 × 10^-18^). We further revealed that the PIN control was significantly higher than that of DMN (*Z* = 6.608; *p* = 3.888 × 10^-11^) and CEN (*Z* = 6.429; *p* = 1.278 × 10^-10^) in MCI group where the AIN was impaired, but that control was significantly lower than of DMN (*Z* = 5.285; *p* = 1.254 × 10^-7^) and CEN (*Z* = 5.404; *p* = 6.513 × 10^-8^) in normal group. Finally, the global cognitive test score assessed using Montreal cognitive assessment and the network modulations were correlated (Spearman’s correlation: *r* = 0.47; *p* = 3.76 × 10^-5^ and *r* = -0.43; *p* = 1.97 × 10^-4^). These findings might suggest the flexible functional profiles of AIN and PIN in normal aging and MCI.

## Introduction

Normal cognitive function involves an effective coordination between functionally associated brain regions or network(s) (Fox et al., 2005; Power et al., 2011). Two core brain networks, namely the *default-mode network* (DMN)—consisting of the posterior cingulate and ventromedial prefrontal cortices, and the *central-executive network* (CEN)—consisting of the posterior parietal and dorsolateral prefrontal cortices, exhibit anti-correlated network activities (Fox et al., 2005; Bressler and Menon, 2010; Chen et al., 2013) with the DMN being more active during internally directed actions while the CEN being more active primarily during externally directed actions (Bressler and Menon, 2010; Uddin, 2015). Recent evidence consistently suggests that this anti-correlation pattern is modulated by the *anterior insula-based network* (AIN), which primarily comprises of the anterior insula and the dorsal anterior cingulate cortex, in both young and elderly people with normal cognition (Sridharan et al., 2008; Chand and Dhamala, 2016a; Wu et al., 2016). We have recently found that the modulation effect of this AIN over the DMN and CEN is impaired in individuals with mild cognitive impairment (MCI) (Chand et al., 2017a,b). Furthermore, recent studies highlight that the insula subdivisions—the anterior insula and the posterior insula—exhibit overlapping profiles/activities that could flexibly involve in a wide range of cognitive processes, especially in restoring the coginitive functions (Starr et al., 2009; Segerdahl et al., 2015; Nomi et al., 2016; Namkung et al., 2017). However, as the modulation ability of the AIN declines in MCI, whether such control feature shifts over to the *posterior insula-based network* (PIN)—network that mainly comprises of the posterior insula and the sensorimotor areas (Deen et al., 2011; Nomi et al., 2016)—has not been previously investigated.

Dynamic interaction analysis between the intrinsic networks has emerged as a potentially valuable approach that may reveal the underlying neural processes in health and disease. Recent functional MRI studies suggest that the AIN is responsible for switching the activation and deactivation between the DMN and CEN in cognitively normal people, and these studies suggest that this control ability maintains individual’s active and passive cognitive states (Menon, 2011; Goulden et al., 2014; Wu et al., 2016). The control functionality of the AIN is reasoned to be carried out with the aid of a unique anatomical cytoarchitectural feature of its key regions—the anterior insula and the dorsal anterior cingulate cortex (Sridharan et al., 2008; Chand and Dhamala, 2016b). Specifically, these regions are anatomically connected (Bonnelle et al., 2012; Jilka et al., 2014) and consist of a special type of neurons named von Economo neurons that are thought to facilitate rapid relays of information from the AIN to the other brain regions such as DMN and CEN (Allman et al., 2005, 2010; Watson et al., 2006; Sridharan et al., 2008). On the other hand, the PIN encompasses the posterior insula and sensorimotor areas, specifically temporal and posterior cingulate regions, and thus are primarily involved in sensorimotor processes (Cauda et al., 2011; Nomi et al., 2016). Structural connectivity analysis consistently demonstrates that posterior insula has direct white matter connections with the parietal and posterior temporal regions (Cerliani et al., 2012; Cloutman et al., 2012; Dennis et al., 2014). Alternation in the AIN activity has been recently reported in the diseases, including autism, frontotemporal dementia, schizophrenia, and MCI or Alzheimer’s disease (Menon, 2011; Uddin, 2015; Chand et al., 2017a,b). Specifically, when the AIN modulation over the DMN and CEN is declined or impaired in MCI, whether the PIN tends to take over this control feature has not been formerly examined.

In the present study, we therefore seek to examine the differential modulation activities of the AIN and of the PIN, respectively, over the DMN and CEN in MCI people and compare with a group of healthy controls. We hypothesized that the control ability of the AIN over the DMN and CEN is disrupted in the MCI group but this control is retained in the healthy normal group. As the AIN preserves this control in the normal group, we further hypothesized that the PIN does not take such control in the normal group, but does tend to take over that control feature in the MCI where the AIN is impaired. We also hypothesized that the global cognitive test score correlates with the modulating probability of the AIN and of the PIN, respectively. To test our hypotheses, we analyzed resting state functional MRI data collected on with a sample of older adults with normal cognition and with the MCI, then applied dynamical causal modeling (DCM), and compared the network interactions between two groups. We also assessed the association between network modulation probability with cognitive performance within the same sample.

## Materials and Methods

### Subjects

This study was carried out in accordance with the recommendations of “Institutional Review Board (IRB) of Emory University” with written informed consent from all subjects. All subjects gave written informed consent in accordance with the Declaration of Helsinki. This study protocol was reviewed and approved by IRB of Emory University. MRI scans were performed on 53 MCI subjects. The MCI subject inclusion criteria were: age ≥ 55 years, hypertension defined by systolic blood pressure ≥ 140 mm Hg or diastolic blood pressure ≥ 90 mm Hg, and MCI assessed based on previously defined criteria (Chao et al., 2009; Pa et al., 2009): Montreal cognitive assessment (MoCA) ≤ 26, cognitive performance at the 10th percentile or below on at least one of four screening tests—trail marking test B, Stroop interference, digit span forward and digit span backward, verbal fluency and abstraction—and minimal functional limitation test as reflected by the functional assessment questionnaire ≤ 7. The subject exclusion criteria were: systolic blood pressure > 200 mm Hg or diastolic blood pressure > 110 mm Hg, renal disease or hyperkalemia, active medical or psychiatric problems, uncontrolled congestive heart failure (shortness of breath at rest or evidence of pulmonary edema on exam), history of stroke in the past 3 years, ineligibility for MRI (metal implants or cardiac pacemaker), inability to complete cognitive test and MRI scan, women of childbearing potential, and diagnosis of dementia (self-reported or care-giver reported). In MCI group, mean age was 66.9 years (*SD*: 8.1), mean education was 15 years (*SD*: 2.4), 60% were African–Americans, 52.8% were women, mean systolic blood pressure 150.7 mm of Hg (*SD*: 21.3), and mean diastolic blood pressure 90.9 mm of Hg (*SD*: 13.5). MRI data were included from 20 normal older adults. The normal control subject inclusion criteria were age ≥ 50 years, MoCA ≥ 26, clinical dementia rating score of 0, and normal logical memory subscale defined as ≥11 for 16 or more years of education, ≥9 for 8–15 years of education, and ≥6 for less than 7 years of education. The exclusion criteria were history of stroke in the past 3 years, ineligibility for MRI (metal implants or cardiac pacemaker), inability to complete cognitive test and MRI scan, and diagnosis of dementia. In cognitively normal group, mean age was 65.8 years (*SD*: 8.8), mean education was 16 years (*SD*: 2.9), 20% were African–Americans, 70% were women, mean systolic blood pressure 128.8 mm of Hg (*SD*: 23.1), and mean diastolic blood pressure 71.7 mm of Hg (*SD*: 11.7), and eight subjects (out of 20) had hypertension. The MCI and normal control groups were not statistically different for age, sex, and education, but were different for systolic blood pressure, diastolic blood pressure, and MoCA score as shown in **Table 1**.

**Table 1:** Mean (standard deviation) of mild cognitive impairment (MCI) group and cognitively normal (NC) group regarding subjects’ age, education, sex, race, systolic and diastolic blood pressures (BP), and Montreal cognitive assessment (MoCA) (*p*-value represents MCI vs. NC comparison using Wilcoxon rank sum test or chi-square test and *p* < 0.05 is considered statistically significant difference between two groups).

Characteristic *N*	MCI group 53	NC group 20	*p*-value
Age, year	66.9 (8.1)	65.8 (8.8)	0.776
Education, year	15 (2.4)	16 (2.9)	0.284
Sex, women	28 (52.8%)	14 (70%)	0.186
Race			
Black	32 (60%)	4 (20%)	
White	19 (36%)	16 (80%)	
Other	2 (4%)		
Systolic BP, mm Hg	150.7 (21.3)	128.8 (23.1)	<10^-3^
Diastolic BP, mm Hg	90.9 (13.5)	71.7 (11.7)	<10^-6^
MoCA	22.3 (3.1)	27.1 (1.1)	<10^-9^


### Image Acquisition

Siemens 3T Trio scanner was used for MRI data acquisition at Center for Systems Imaging of Emory University. Anatomical 3D images were acquired using T1-weighted magnetization prepared rapid gradient echo (MPRAGE) sagittal with the repetition time (TR) = 2300 ms, echo time (TE) = 2.89 ms, inversion time (TI) = 800 ms, flip angle (FA) = 8°, resolution = 256 × 256 matrix, slices = 176, and thickness = 1 mm. Resting state blood oxygenation level dependent (BOLD)-fMRI images were acquired axially using an echo-planar imaging sequence with the TR = 2500 ms, TE = 27 ms, FA = 90°, field of view = 22 cm, resolution = 74 × 74 matrix, number of slices = 48, thickness = 3 mm and bandwidth = 2598 Hz/Pixel. The subjects were asked to hold still, keep their eyes open, and think nothing during scan time.

### Image Preprocessing

MRI images were preprocessed using SPM12 (Wellcome Trust Centre for Neuroimaging, London, United Kingdom^1^). The preprocessing included slice-timing correction, motion correction, co-registration to individual anatomical image, normalization to Montreal Neurological Institute template, and spatial smoothing of normalized images using a 6 mm isotropic Gaussian kernel. Independent component analysis (ICA) was carried out on the preprocessed data.

### Independent Component Analysis

Independent component analysis is a promising technique for the functional brain activities. A spatially constrained ICA (Lin et al., 2010) has been proposed for the study of specific brain areas or networks. In this work, we used the templates of DMN, AIN, PIN, and CEN from previous study (Shirer et al., 2012) in Group ICA of fMRI Toolbox (GIFT^2^) and computed ICA component of each network. Prior studies suggest that ICA component of each network/mask is more accurate than the average or first eigen-variate of network template/mask (Smith et al., 2011; Craddock et al., 2012; Shirer et al., 2012; Goulden et al., 2014). We first ran ICA analysis separately for the normal controls and MCI. We subsequently implemented a DCM on the ICA-components of networks. For cross-validation purpose, we also ran ICA analysis combinely for the normal controls and MCI and then implemented DCM.

### Dynamical Causal Modeling

Dynamical causal modeling (Friston et al., 2003) infers the statistical measure of directed functional connectivity between brain areas or networks. DCM bases on Bayesian model selection and compares the user defined models with the measured data (Stephan et al., 2010). DCM has recently been implemented in resting state fMRI (Daunizeau et al., 2012; Friston et al., 2014).

In model construction, DCM models were designed with full intrinsic connections between the networks and the modulations were taken to represent the models. In DCM analysis, *model 1* represents non-linear modulation of DMN on both connections between AIN (or PIN) and CEN. Similarly, *model 2* represents non-linear modulation of AIN (or PIN) on the connections between DMN and CEN, and *model 3* represents non-linear modulation of CEN on the connections between AIN (or PIN) and DMN. We performed both fixed effect and random effect Bayesian model selection methods. In brief, a fixed effect considers that the optimal model is homogeneous across subjects and provides the group log-evidence that measures the balance between fit and complexity of models and quantifies the relative goodness of models. On the other hand, a random effect accounts for heterogeneity of model structure across subjects and provides the posterior model probability, which measures how likely a specific model generated the data of randomly selected subject, and the posterior exceedance probability that measures how one model is more likely than any other model (Stephan et al., 2010). DCM analysis was performed by using SPM12 (Wellcome Trust Centre for Neuroimaging, London, United Kingdom^1^).

### Statistical Analysis

Network modulation probabilities were compared between cognitively normal group and MCI group using Wilcoxon rank sum test. Correlation analysis was performed between the global neuropsychological test score assessed by MoCA and the modulation probability of AIN and/or PIN to the DMN and CEN using Spearman’s correlation. Matlab software (Natick, MA, United States^3^) was used to analyze the data.

## Results

### Constrained ICA

**FIGURE 1:**
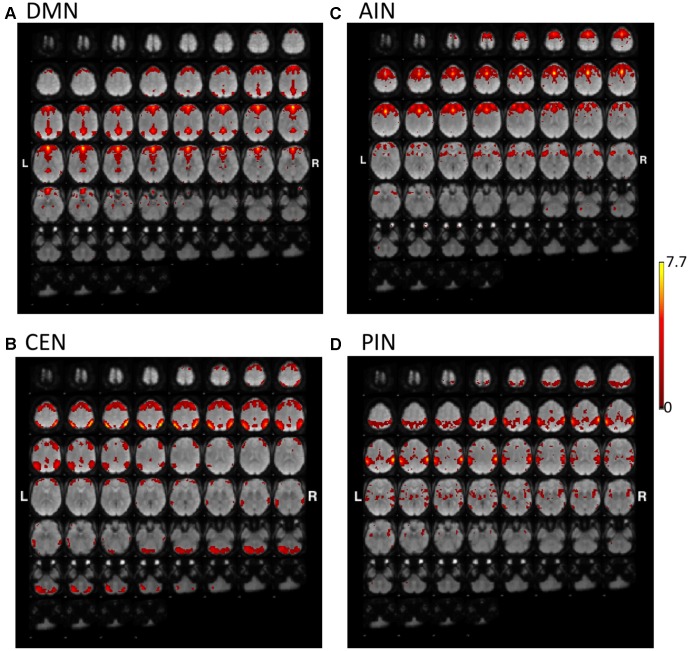
The t-value maps of **(A)** default-mode network (DMN), **(B)** central-executive network (CEN), **(C)** anterior insula-based network (AIN), and **(D)** posterior insula-based network (PIN) from the constrained ICA overlaid on mean BOLD images in the normal control group (L and R indicate the left and right hemispheres, respectively).

**FIGURE 2:**
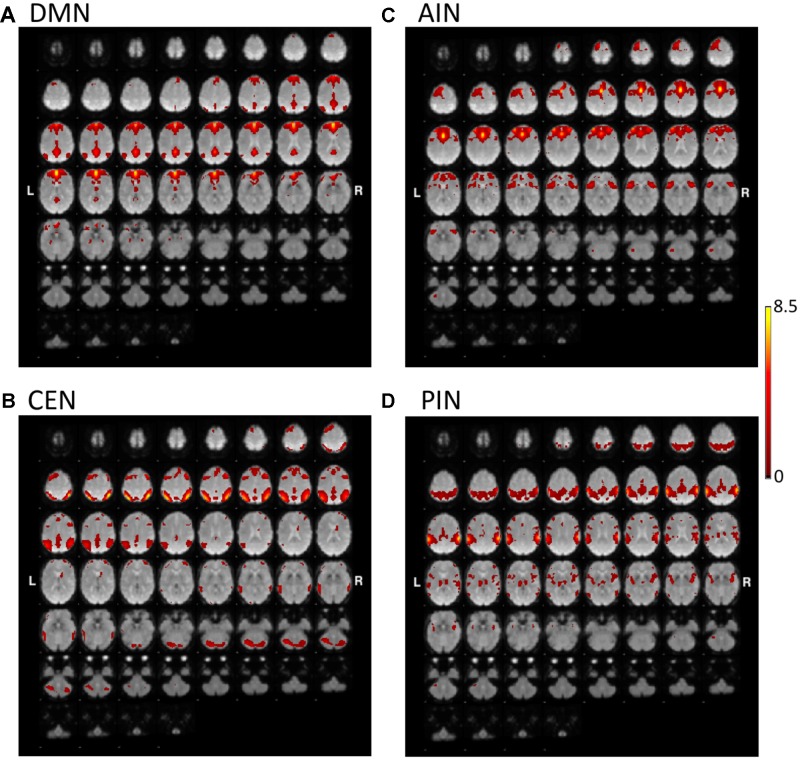
The t-value maps of **(A)** DMN, **(B)** CEN, **(C)** AIN, and **(D)** PIN from the constrained ICA overlaid on mean BOLD images in mild cognitive impairment (MCI) (L and R indicate the left and right hemispheres, respectively).

**Figures 1**, **2** show the results of constrained ICA of DMN, CEN, AIN, and PIN for the normal control group and the MCI group, respectively.

### DCM Model Comparisons

**FIGURE 3:**
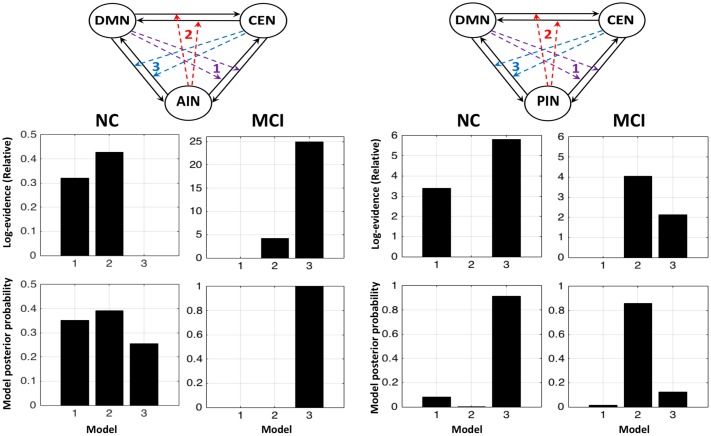
Fixed effects results for the normal controls (NCs) and the MCI in terms of log-evidence and posterior probability. The first and second columns display that the modulations by AIN over central-executive and default-mode networks (CEN, DMN) had a higher probability than other models in the NC but not in the MCI, respectively. The fourth and third columns display that the modulations by PIN had a higher probability in the MCI but not in the NC, respectively.

**FIGURE 4:**
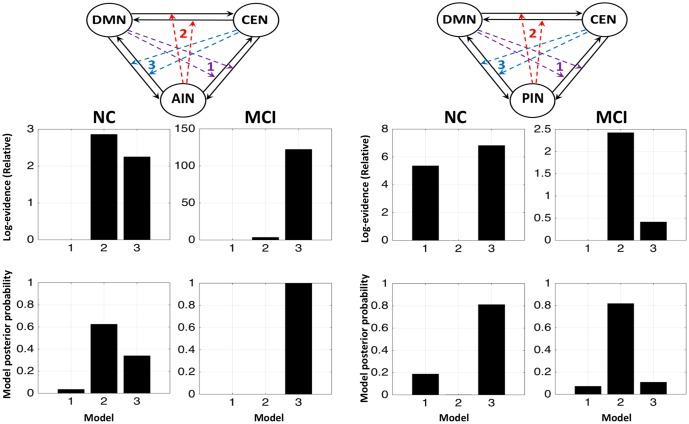
Fixed effects results for the NCs and the MCI by running ICA combinely in two groups. The first and second columns display that the modulations by AIN over central-executive and default-mode networks (CEN, DMN) had a higher probability than other models in the NC but not in the MCI, respectively. The fourth and third columns display that the modulations by PIN had a higher probability in the MCI but not in the NC, respectively.

**Figure 3** shows the fixed effect results for normal controls and MCI expressed in terms of log-evidence and posterior probability. Fixed effect method for the normal control showed that a control feature by AIN (model 2: first column) has higher probability compared with that of control feature by DMN (model 1: first column) and by CEN (model 3: first column). Fixed effect method further demonstrated that the control feature of AIN (model 2: second column) no longer has dominant probability in the MCI. We further investigated the control feature of PIN in the normal control (model 2: third column) and in the MCI (model 2: fourth column). In the normal control, we found that the model 2 (control feature of PIN) does not have dominant switching probability. In the MCI, we uncovered that the model 2 (control feature of PIN) came in play and possessed the higher probability compared with that of models 1 and 3. DCM analysis was also carried out by running ICA together in normal controls and MCI (**Figure 4**). We found the similar patterns of higher model probability. The AIN modulation has higher probability in the normal controls, but not in the MCI (model 2 in the first and second columns). Moreover, the PIN modulation did not have higher probability in the normal controls, but had higher probability than other models in the MCI (model 2 in the third and fourth columns).

**FIGURE 5:**
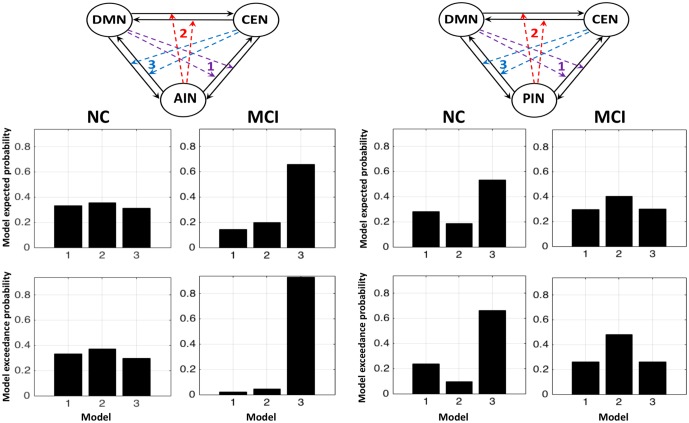
Random effects results for the NCs and the MCI in terms of expected and exceedance probabilities. The first and second columns display that the modulations by AIN over central-executive and default-mode networks (CEN, DMN) had a higher probability than other models in the NC but not in the MCI, respectively. The fourth and third columns display that the modulations by PIN had a higher probability in the MCI but not in the NC, respectively.

We further performed the random effect analysis. **Figure 5** displays the random effect results for the normal control and the MCI expressed in terms of expected and exceedance probabilities. In the normal control, we found that a control feature of AIN (model 2: first column) has higher probability compared with that of control feature of DMN (model 1: first column) and of CEN (model 3: first column). In the MCI, we further revealed that the control feature of AIN (model 2: second column) no longer has dominant probability. We also examined that the control feature of PIN in the normal control (model 2: third column) and the MCI (model 2: fourth column). We found that control feature of PIN (model 2) does not have dominant probability in the normal control, but it has higher probability than other models in the MCI group. The modulation probability from PI to AI increased in MCI (Supplementary Figure S1).

**FIGURE 6:**
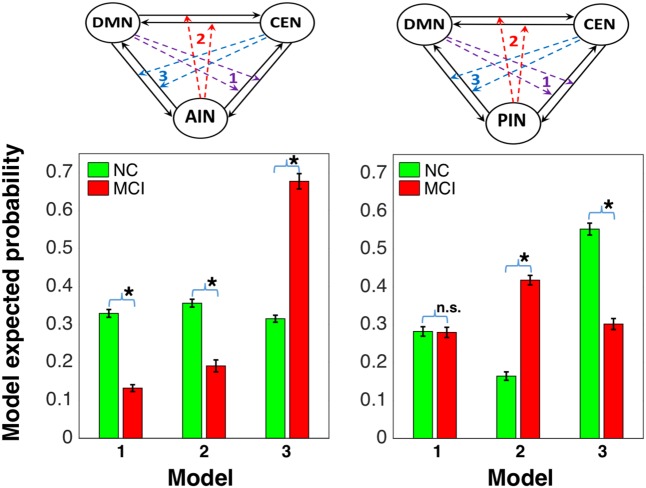
Model comparison between NC and MCI groups: the first column shows that AIN modulation (represented by model 2 in the left side) is higher than the modulations by default-mode and central-executive networks (DMN and CEN) in NC and it is declined in MCI, and the second column shows that the PIN modulation (represented by model 2 in right side) is not higher in NC and it is elevated in MCI [^∗^indicates statistical significance with *p* < 0.05 (false discovery rate-corrected) and n.s. indicates statistically not significant].

We compared the model probability within the group and between the groups using Wilcoxon rank sum test. In normal control group, we found that modulation by AIN (model 2 in left column **Figure 6**) had statistically higher probability than model 1 (*p* = 0.046; *Z* = 1.987) and model 3 (*p* = 0.002; *Z* = 3.076), but there was no statistical difference between model 1 and model 3 (*p* = 0.531; *Z* = 0.627). In MCI group, AIN did not have higher probability, but model 3 had significantly higher probability than model 1 (*p* = 8.539 × 10^-19^; *Z* = 8.853) and model 2 (*p* = 7.816 × 10^-18^; *Z* = 8.602) and model 2 had higher probability than model 1 (*p* = 1.911 × 10^-4^; *Z* = 3.731). Each model probability showed statistical difference between normal control and MCI groups: model 1 (*p* = 8.606 × 10^-11^; *Z* = 6.489), model 2 (*p* = 8.257 × 10^-9^; *Z* = 5.763), and model 3 (*p* = 4.089 × 10^-10^; *Z* = 6.251). On the other hand, in normal control group we found that modulation by PIN (model 2 in right column) did not have higher probability. Model 3 had significantly higher probability than model 1 (*p* = 7.616 × 10^-8^; Z = 5.376) and model 2 (*p* = 6.513 × 10^-8^; *Z* = 5.404) and model 1 had higher probability than model 2 (*p* = 1.254 × 10^-7^; *Z* = 5.285). In MCI group, our analysis revealed PIN modulations (model 2 in right column) had statistically higher probability than model 1 (*p* = 3.888 × 10^-11^; *Z* = 6.608) and model 3 (*p* = 1.278 × 10^-10^; *Z* = 6.429), but there was no statistical difference between model 1 and model 3 (*p* = 0.197; *Z* = 1.289). In PIN modulations, there was statistical difference between normal control and MCI groups in model 2 (*p* = 5.216 × 10^-10^; *Z* = 6.212) and in model 3 (*p* = 1.557 × 10^-9^; *Z* = 6.038), but not in model 1 (*p* = 0.985; *Z* = 0.019).

### Association between Network Interactions and Cognitive Scores

**FIGURE 7:**
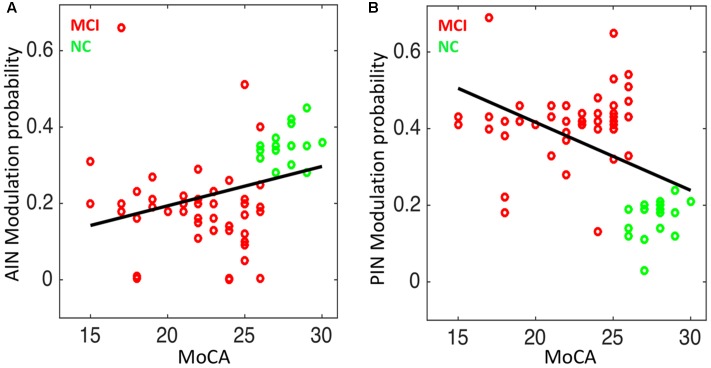
Correlation analysis in NC and MCI: **(A)** the modulation probability of AIN over the default-mode and central-executive networks (DMN and CEN) versus the MoCA score (Spearman’s correlation: *r* = 0.47; *p* = 3.76 × 10^-5^), and **(B)** the modulation probability of PIN over the DMN and CEN versus the MoCA score (*r* = –0.43; *p* = 1.97 × 10^-4^).

We studied the association between the network modulation probability and the cognitive scores. We found statistically significant correlation between the MoCA and the modulation probability of AIN (Spearman’s correlation: *r* = 0.47; *p* = 3.76 × 10^-5^) and of PIN (*r* = -0.43; *p* = 1.97 × 10^-4^), respectively, as shown in **Figure 7**.

**FIGURE 8:**
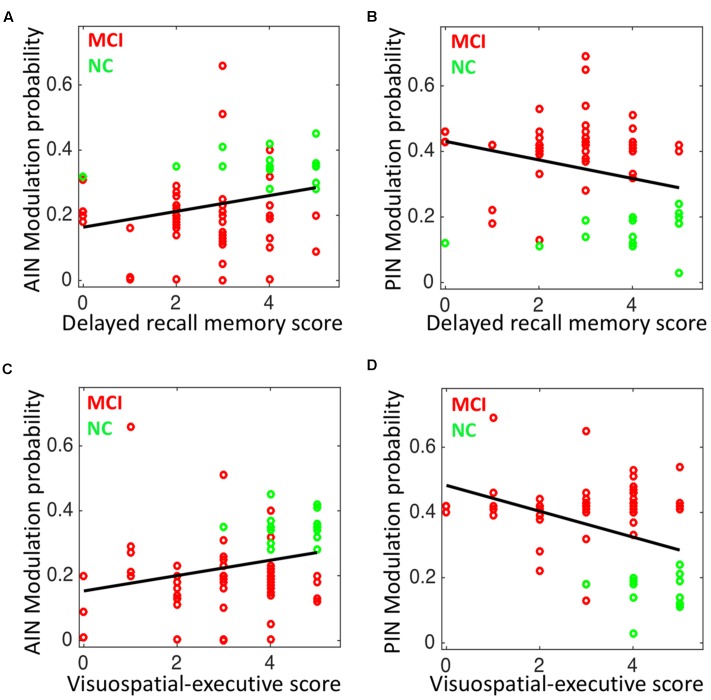
Correlation analysis in NC and MCI: **(A)** the modulation probability of AIN over the default-mode and central-executive networks (DMN and CEN) versus the delayed recall memory function subscore of MoCA (Spearman’s correlation: *r* = 0.30; *p* = 0.010), **(B)** the modulation probability of PIN over the DMN and CEN versus the delayed recall memory function subscore of MoCA (*r* = –0.299; *p* = 0.012), **(C)** the modulation probability of AIN over the DMN and CEN versus the visuospatial-executive function subscore of MoCA (*r* = 0.343; *p* = 0.004), and **(D)** the modulation probability of PIN over the DMN and CEN versus the visuospatial-executive function subscore of MoCA (*r* = –0.304; *p* = 0.011).

The modulation probability significantly associated with the delayed recall memory function and visuospatial-executive function “Subscores of MOCA”. Delayed recall memory test score associated with the modulation probability of AIN (Spearman’s correlation: *r* = 0.30; *p* = 0.010), and with the modulation probability of PIN (*r* = -0.299; *p* = 0.012). Visuospatial-executive test score correlated with the modulation probability of AIN (*r* = 0.343; *p* = 0.004), and with the modulation probability of PIN (*r* = -0.304; *p* = 0.011) (**Figure 8**). Trail marking test B score showed weak correlation with the modulation probability of AIN (*p* = 0.173) and of PIN (*p* = 0.058).

## Discussion

Here, we evaluated the switching/modulation effects of insula subdivisions-based networks—AIN and PIN—on the DMN and CEN in MCI group in comparison to a group of normal controls. The AIN was found to exert modulation effects on the DMN and CEN in control group, consistent with former studies (Sridharan et al., 2008; Chand and Dhamala, 2016a; Wu et al., 2016). However, this modulation effect of the AIN was impaired in MCI group (Chand et al., 2017a). Furthermore, the PIN did not provide modulation effects on the DMN and CEN in normal group, and in contrast the PIN took over some control feature of the AIN in MCI group (the AIN was impaired in MCI). Finally, the global cognitive test scores were correlated with the modulating probability of the AIN and of the PIN.

Previous investigations suggest that control feature of AIN in cognitively normal group might be carried out with the help of Von Economo neurons (Allman et al., 2005, 2010; Watson et al., 2006). Specifically, those studies report that Von Economo neurons are present abundantly in anterior insula and dorsal anterior cingulate cortex nodes of AIN, but there are no reports of their presence in posterior insula and sensorimotor area of PIN. Literature shows that the anterior insula of AIN is functionally connected to the networks responsible for adaptive behavior (Seeley et al., 2007) and to the fronto-parietal control network (Vincent et al., 2008). Anterior insula also has a direct white matter connections to other key brain nodes and lobes such as dorsal anterior cingulate cortex (Jilka et al., 2014), inferior-parietal lobe (Uddin et al., 2010), and the temporo-parietal junction (Kucyi et al., 2012). Thus, the anterior insula involves in a wide range of cognitive processes, including reorienting the attention (Ullsperger et al., 2010) and switching between cognitive resources (Uddin and Menon, 2009). The activity in the dorsal anterior cingulate cortex of AIN is crucial in monitoring the conflict, switching between cognitive states in association with anterior insula during harder decision-making tasks, and implement behavioral changes (Egner, 2009; Chand and Dhamala, 2016a). The control signal of AIN might be carried out by the neural bases mentioned above. The PIN encompasses the posterior insula and sensorimotor areas, specifically temporal and posterior cingulate regions, and is suggested to involve in interoceptive and/or sensorimotor processes (Cauda et al., 2011; Nomi et al., 2016). The posterior insula has well-developed functional connections with the auditory cortex and has been consistently reported in auditory processing, supporting the findings that it is mainly a sensory region (Cauda et al., 2011). Structural connectivity analysis consistently demonstrates that posterior insula has direct white matter connections with the parietal and posterior temporal regions, and anterior temporal regions to a lesser extent (Cerliani et al., 2012; Cloutman et al., 2012; Dennis et al., 2014). The posterior insula and the middle insula are consistently reported to exhibit overlapping cognitive functions (Deen et al., 2011). Emerging studies report that the insula subdivisions exhibit the unique and overlapping profiles in a wide range of cognitive processes and argue that such overlapping functional profiles might be helpful in restoring cognitive functions (Starr et al., 2009; Segerdahl et al., 2015; Nomi et al., 2016; Namkung et al., 2017). The control ability achieved by the PIN in MCI in the present study might thus support the putative roles of overlapping functional activities of the insula divisions.

Literature reports that the AIN atypically engaged in disease, including autism, schizophrenia, fronto-temporal dementia, and Alzheimer’s disease (Menon, 2015; Uddin, 2015; Chand et al., 2017a,b). A large body of MCI and/or Alzheimer’s disease investigations repeatedly suggest that the DMN activity decreases (Greicius et al., 2004; Greicius and Kimmel, 2012; Brier et al., 2014), but the role of CEN activity has been conflicted with the progression of disease (Diener et al., 2012). The CEN, especially its dorsolateral prefrontal cortex node, abundantly connects with visual, somatosensory, and auditory areas, and therefore might possess the crucial role in a wide range of cognitive functions, including goal-orientated actions (Petrides and Pandya, 1999; Miller and Cohen, 2001; Chand et al., 2016; Chand and Dhamala, 2017). The functional role of CEN activity has been inconsistently reported in disease (Diener et al., 2012). Whether the dorsolateral prefrontal cortex—a key node of CEN—hyperactive or hypoactive in disease has remained conflicting in those studies. In our case, we observed the higher probability of CEN modulation with the AIN and DMN in MCI. On the other hand, the modulation probability of CEN during interactions with the PIN and DMN was smaller in MCI than in normal control group. The alterations of CEN modulations thus remain unclear. Prior studies and our findings together suggest that, as AIN control is disrupted in MCI individuals, the PIN might come up to take over the control features, and this control might possibly decline when MCI changes to dementia or Alzheimer’s disease. A detailed description of this decline mechanism can be explored in the future by including the data from individuals with dementia or Alzheimer’s disease.

In summary, we evaluated the patterns of connectivity of the PIN and/or AIN over the DMN and CEN in MCI people and compared with a group of cognitively normal people. We revealed that the PIN took control over DMN and CEN in MCI group where the control activity of AIN was impaired. These findings provide important implications about the underlying flexible functional profiles of insula subdivision-based brain networks and their interactions in normal cognition and MCI.

## Disclosure Statement

All authors have approved the manuscript and agree with submission to this journal. We have read and have abided by the statement of ethical standards for manuscripts submission.

## Author Contributions

Designed the experiment: IH and DQ. Performed the experiment: GC, JW, DQ, and IH. Analyzed the data: GC, DQ, and IH. Wrote the paper: GC, DQ, and IH. Participated in the discussion and provided the comments: GC, JW, DQ, and IH.

## Conflict of Interest Statement

The authors declare that the research was conducted in the absence of any commercial or financial relationships that could be construed as a potential conflict of interest.

## Footnotes

1 www.fil.ion.ucl.ac.uk/spm/software/spm12

2 http://mialab.mrn.org/software/gift

3 https://www.mathworks.com
