# Supplementary material for: Interactions of Insula Subdivisions-Based Networks with Default-Mode and Central-Executive Networks in Mild Cognitive Impairment
Source: Front Aging Neurosci. 2017 Nov 9;9:367. doi: 10.3389/fnagi.2017.00367 (PMC5684105; doi:10.3389/fnagi.2017.00367)
Supplement: Supplementary file 1 [file Data_Sheet_1.docx]

**Supplementary Figure**


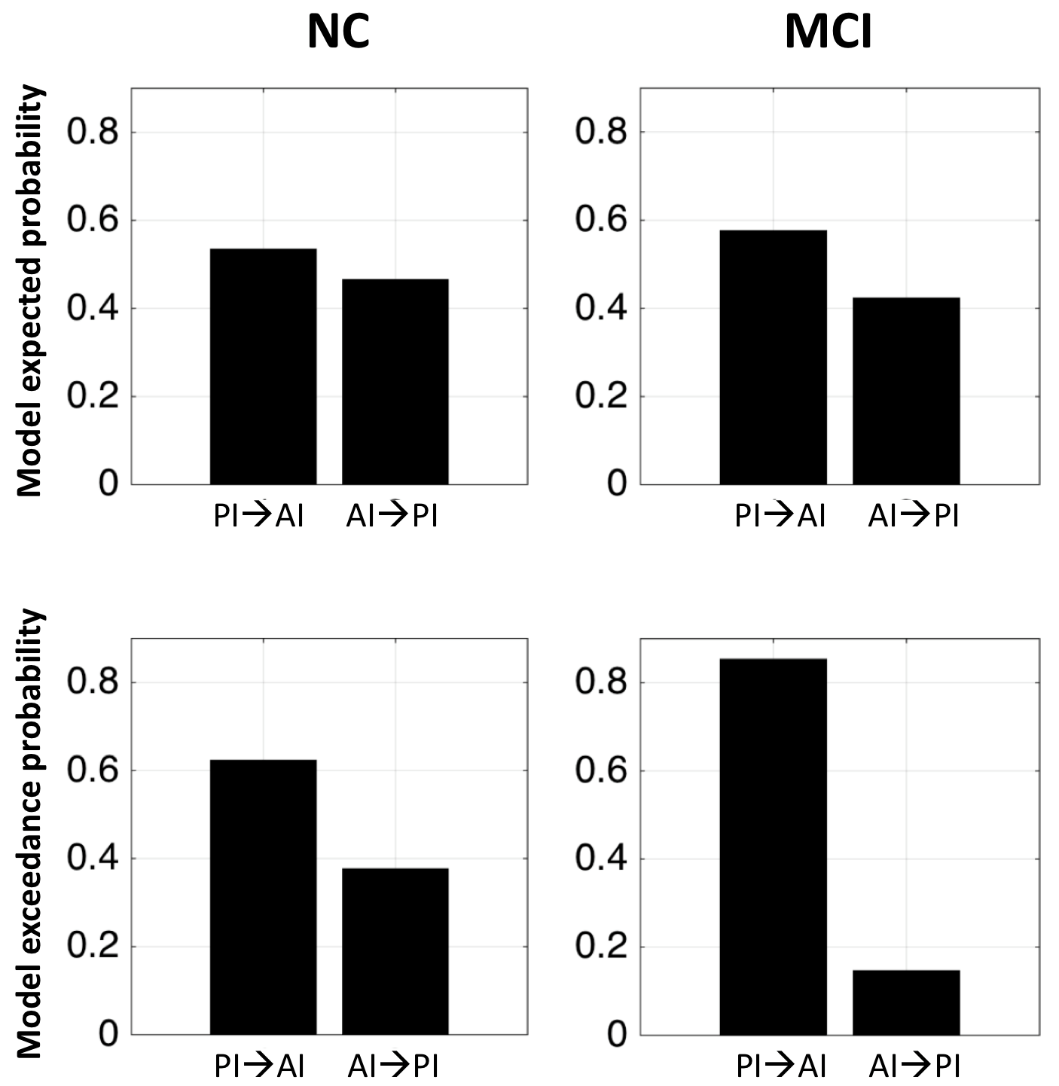


**Fig S1:** DCM results between the posterior insula (PI) and anterior insula (AI) for the normal controls (NC) and the mild cognitive impairment (MCI) demonstrating the increase in modulation probability from the posterior insula to the anterior insula (and/or, the decrease in modulation probability from the anterior insula to the posterior insula) in MCI than that in NC.
